# Adaptive Data Gathering in Mobile Sensor Networks Using Speedy Mobile Elements

**DOI:** 10.3390/s150923218

**Published:** 2015-09-15

**Authors:** Yongxuan Lai, Jinshan Xie, Ziyu Lin, Tian Wang, Minghong Liao

**Affiliations:** 1 School of Software, Xiamen University, 422 Siming South Road, Siming District, Xiamen 360000, China; E-Mail: liao@xmu.edu.cn; 2 School of Mathematics and Computer Science, Longyan University, Longyan 364000, China; E-Mail: jsxie@lyun.edu.cn; 3 Department of Computer Science, Xiamen University, 422 Siming South Road, Siming District, Xiamen 360000, China; E-Mail: ziyulin@xmu.edu.cn; 4 College of Computer Science and Technology, Huaqiao University, Xiamen 360000, China; E-Mail: wangtian@hqu.edu.cn

**Keywords:** proxy node selection, time slot allocation, data gathering, mobile sensor network

## Abstract

Data gathering is a key operator for applications in wireless sensor networks; yet it is also a challenging problem in mobile sensor networks when considering that all nodes are mobile and the communications among them are opportunistic. This paper proposes an efficient data gathering scheme called ADG that adopts speedy mobile elements as the mobile data collector and takes advantage of the movement patterns of the network. ADG first extracts the network meta-data at initial epochs, and calculates a set of proxy nodes based on the meta-data. Data gathering is then mapped into the Proxy node Time Slot Allocation (PTSA) problem that schedules the time slots and orders, according to which the data collector could gather the maximal amount of data within a limited period. Finally, the collector follows the schedule and picks up the sensed data from the proxy nodes through one hop of message transmissions. ADG learns the period when nodes are relatively stationary, so that the collector is able to pick up the data from them during the limited data gathering period. Moreover, proxy nodes and data gathering points could also be timely updated so that the collector could adapt to the change of node movements. Extensive experimental results show that the proposed scheme outperforms other data gathering schemes on the cost of message transmissions and the data gathering rate, especially under the constraint of limited data gathering period.

## 1. Introduction

Recent years there have been a lot of applications in Wireless Sensor Networks (WSNs), ranging from monitoring to event detection and target tracking. For all these applications, data gathering is one of the primary operations carried out in WSNs [1,2,3,4]. Traditionally, the network is assumed to be dense so that there are end-to-end multi-hop paths within the network, along which the generated data could be routed to the base station. This assumption, however, does not always hold in the scenarios of real network deployments. For example, as the WSN is often deployed in harsh environments, the signal is susceptible to external interference and leads to disconnected and portioned network; and if the network is sparse or the nodes are mobile, the paths to the sink might not always be available. So recently there is a research trend that adopts mobile elements for the message transmission and data gathering in mobile sensor networks [5,6].

In these schemes mobile data collectors, e.g., autonomous robots, are used to move within the sensing field, collects data from sensor nodes and brings them to the sink. The ordinary nodes, on the other hand, just stay stationary and wait for the mobile data collector to come and pick up the data. So the *funnelling effect* [7], where the energy of nodes near the sink is depleted quickly because of forwarding more data packets than nodes distant from the sink, could be avoided. Mobile elements also make data gathering in a sparse or disconnected network possible because the collector can travel and directly collect the data from sensors. One strategy of data gathering with mobile elements is to map the data gathering problem into the optimization of trajectories of the mobile collector, and various scheduling schemes are proposed to increase the network lifetime or to minimize the traveling distance of mobile elements [1,3,4,6,8,9,10]. In these schemes, however, ordinary nodes should be stationary and there should be paths or tracks available among the nodes, which are not always true in network deployments. Another strategy [11,12,13,14] is to adopt an opportunistic “Store-Carry-Forward” routing protocols, the same as those in the Delay-Tolerant Networks [15,16], for data gathering when both the ordinary nodes and data collector are mobile. Because both the ordinary nodes and the collector are mobile, there is no stable trajectories along which the collector could move within the network for data gathering, neither are the static links between the source node and the collector. So a *flooding* or *epidemic* approach is usually adopted for the message routing, which leads to unnecessary transmissions and energy depletion of nodes.

In this paper, we study the problem of data gathering in mobile sensor networks with speedy mobile elements, especially when there is no predefined paths or tracks for the data collector. For example, an autonomous underwater vehicle is used as a mobile sink to gather data from a randomly distributed underwater sensor network [17]; in the wildlife monitoring application [11], sensor nodes are attached to the monitoring targets so that they could opportunistically exchange messages and upload the sensed data to the data collector. Yet at these scenarios the “on-land” data collector, e.g., SenCar [8], is usually not suitable for the data gathering because the collector moves relatively at low speed, and there might not be any path or track available for the data collector due to the harsh environment of the sensing field. In contrast, a speedy and track-free data collector, e.g., a modeling aircraft actuated by a remote controller or unmanned aerial vehicle, could move to any place within the network as well as to be able to stay stationary at any location. It is ideal to be used as a data collector, and recent events, e.g., Fukushima nuclear reactor explosion, have highlighted the need for unmanned remote sensing in dangerous areas, particularly where structures have collapsed or explosions have occurred [18]. The main concern is that the period of data gathering is shorter, because the speedy data collector has a higher energy consuming rate and has to replenish its energy after a round of trip.

This paper proposes an efficient data gathering scheme called ADG at mobile wireless sensor networks, which belongs to the category of mobile ad hoc network (MANETs). All nodes are mobile and ad hoc. The movements and sparse deployment of nodes usually lead to intermitted connected links and create some form of opportunistic communications. Also, the data gathering is assumed to be delay-tolerant as there is some delay for the mobile data collector to visit the in-network nodes, pick up data from them and then go back to the base station for data uploading. The idea behind ADG is that with the advancement of mechanical and electronic technologies, a speedy and track-free mobile node could be used as a data collector for the purpose of data gathering, and through the collection of network meta-data, the rough moving pattern of nodes could be adopted to guide the data collector. The collector could then be programmed to move to the optimized locations to collect the sensing data through one hop transmissions, which avoids lots of transmissions and greatly improves the overall performance. Also, as the nodes are mobile, ADG also learns the period when nodes are relatively stationary, so that the mobile collector (MC) is able to pick up the data from them during the limited data gathering period. The main characteristics of the proposed scheme lies in the following aspects:

- It is the first step on the research of data gathering using speedy mobile elements in mobile sensor networks within a limited data gathering period, while most of the existing research focuses on data gathering on stationary sensor network. MC is assumed to move faster in ADG, yet with much smaller time length within a data gathering round. So the contact opportunities with other ordinary mobile nodes are fewer, shorter, and more opportunistic in nature, which makes the data gathering a challenging problem;
- The scheme does not need predefined paths or tracks for the data gathering, while other schemes [4,8,9,19] do need compute a track or path for MC. The dynamic nature of mobile networks makes it impossible for the MC to move along a precomputed path for the data gathering. Instead, ADG calculates a set of proxy nodes that act as an intermediate storage to receive sensed data from ordinary nodes, which would make it efficient for the MC to collect the sensed data;
- It maps the data gathering into the a variant of the Knapsack problem [20], the target of which is to maximize the expected amount of gathered data under the constraints of a compatible schedule and a limited data gathering period. ADG would schedule the time slots and orders to gather as much data as possible. Our work is orthogonal to the compressive sensing techniques, e.g., sub-nyquist sampling [21], which could be integrated into our scheme to reconstruct images or signals accurately from far smaller data size than the desired resolution of the image/signal.

Extensive experimental results show that the proposed algorithm outperforms other epidemic and probabilistic data gathering algorithms on the overhead of message transmissions and the data gathering rate, especially under the constraint of limited data gathering period. The rest of paper is structured as follows: Section 2 surveys some existing research related to this paper; Section 3 gives some assumptions about the network model; Section 4 describes the detailed mechanism of the proposed scheme ADG, including the meta-data extraction, proxy node selection, time slot allocation, and data gathering from proxy nodes. Finally, Section 5 describes the experimental setup and performance evaluation, and Section 6 concludes the paper.

## 2. Related Work

Wireless sensor network is *data-oriented* as every node might generate some data, and data gathering is indeed a broad topic in the field of wireless sensor network [2,22]. Most the data gathering research focus on either the energy efficiency or smaller amount of data gathered, and they usually depend on infrastructures such as query trees or clustering to collect data. Wei *et al*. [23] proposed a prediction-based data collection protocol in which a double-queue mechanism is designed to synchronize the prediction data series of the sensor node and the sink node. The results showed the approach reduced communication redundancy and improved the lifetime of wireless sensor networks. Xi *et al*. [24] proposed a hierarchical data aggregation method using compressive sensing that combines a hierarchical network configuration. The model was showed to guarantee accurate signal recovery performance and provide substantial energy savings. Yao *et al*. [22] proposed both a centralized heuristic to reduce its computational overhead and a distributed heuristic to make the data gathering algorithm scalable for large-scale network operations.

In mobile wireless sensor networks, it is expensive to maintain these infrastructures, so infrastructure-free strategies with mobile elements are adopted in the data gathering algorithms. In this section we survey some related work of data gathering schemes in sensor networks using mobile elements. Clearly, the mobility pattern of the mobile element significantly impacts the optimal data collection scheme; so we roughly classify the gathering schemes into three categories according to the type of node movements: *trajectory based, roaming based*, and *opportunistic based*.

In *trajectory based* scheme, one or more collectors are scheduled to periodically move along a track and collect the data. Shah *et al*. [1] proposed the DataMULEs system, where the collector (MULE node) collects the sensing data and routes them to the access point through one or multiple hops of transmissions. Wang *et al*. [3] proposed a method for using a mobile sink for data collection and increasing network lifetime. It uses a linear optimization model to determine the collector’s trajectory: which nodes should be visited, and how long the sojourn time should be. Gu *et al*. [25] defined a trajectory as a closed polygonal chain and derived the trajectory of the collector in different phases. At the partitioning phase, nodes are grouped based on distance and buffer overflow times; during the scheduling phase, paths within each group are calculated as solutions to the Traveling Salesman Problem (TSP), and the group paths are concatenated to obtain the complete trajectory in the network. In this way, it avoids message loss at sensors due to buffer overflows. Ma and Yang [4] proposed a moving path planning algorithm by finding some turning points. The algorithm is adaptive to the sensor distribution and can effectively avoid obstacles on the path, and sensors would forward packets to the mobile collector along each moving line segment in a multi-hop fashion. Zhao and Yang [8] extended the work in [4], they formalized the problem as two convex optimization problems aiming to maximize the overall network utility while guaranteeing the given network lifetime and data gathering latency. The scheme considers cases when the collector spends fixed and variable sojourn time at each anchor point, and involves the joint design of rate control, optimal routing, data control, and sojourn time allocation problems. Xu *et al*. [9] proposed a scheme that adopts the mobility of the sink node and the spatial-temporal correlation of the event for data gathering. Data gathering is modeled as a sensor selection problem, and it designed a feasible moving route for the mobile sink to maximize the network lifetime at a guaranteed event collection rate and to minimize the velocity requirements for a practical system. Similarly, Zhao and Yang [10] proposed a polling-based mobile gathering approach where a subset of sensors will be selected as polling points that buffer the aggregated data locally and upload the data to the mobile collector when it arrives. They formulated the problem into an optimization problem of bounded relay hop mobile data gathering. More recently, Van Le *et al*. [6] proposed a hierarchical data gathering scheme that used two types of mobile elements: the mobile collector (MC) and the mobile relay (MR). MC’s collect data from sensors and forward them to the MR, which will deliver them to the sink. It formulated the problem as an integer linear programming (ILP) optimization problem aiming to find the optimal trajectories for MC’s and the MR such as to minimizing the traveling distance of mobile elements.

The *roaming based* schemes are similar to the trajectory based schemes, which also have a trajectory or track. However, in trajectory based schemes the collector strictly follows the trajectory once the trajectory is scheduled or calculated; while in roaming based schemes the mobile collector might roam away from its track or trajectory due to special events or constraints, such as buffer overflow or latency due to data collection. The collector could freely move to any location in the field to collect the data. Zhao *et al*. [26] proposed a *node-initiated message ferrying* approach. When an event is detected by the stationary nodes, the nodes would send a request to the the collector in order to be visited; the collector could modify its trajectory by visiting the requesting node, and then go back to the original route. Gu *et al*. [19] introduced the *mobile element scheduling* problem where nodes operate with different sampling rates. They formulated the problem of scheduling the mobile element in the network to prevent buffer overflows at source nodes. The problem is shown to be NP-complete and an integer-linear-programming formulation is given. Campbell *et al*. [27] extended the work in [25] and proposed a scheme that differentiates message delivery considering both regular and urgent message collection. They incorporated multi-hop communication into the mobile element scheduling problem, where the investigated performance metrics include the minimum required speed of mobile element to prevent data loss and guarantee the maximum tolerated urgent message delay, and the urgent and regular message loss rates for a given speed.

In *trajectory* and *roaming* based data gathering schemes, ordinary nodes are stationary. They just wait the collector to come and pick up the data. Yet, in *opportunistic* data gathering schemes both the data collector and the ordinary nodes are mobile, and an optimized trajectory is very expensive to maintain and is usually impossible when designing the data gathering schemes [13]. Examples of these network have been successfully employed in the context of wildlife monitoring applications, such as tracking of zebras in the ZebraNet project [11] or whales in the SWIM system [12]. Sensor nodes are attached to animals and act as peers, and adopt a “Store-Carry-Forwarding” strategy for message transmission. Based on the history of node movements, each node maintains a probability to the sink, and the node with higher probability would forward its messages to the node with lower probability if there exists chance of communication at some proper time. When mobile peers get close to a base station, the gathered data is uploaded to the base station, and is flushed by peers in order to save storage. Also, the concept of *people sensing* is introduced for opportunistic data collection through mobile peers in urban sensing scenarios [14,28]. Sensors are not used mainly for monitoring the environment, but are rather exploited to characterize people in terms of both interactions and context (or state) information. Sample applications include personal monitoring (e.g., physical exercise tracking), civil defense (e.g., hazards and hotspot reporting to police officers) and collaborative applications (e.g., information sharing for tourism purposes). Ayaki *et al*. [29] proposed a data gathering scheme in urban streets using mobile phones as the relayed nodes. Relay nodes that roam around the area receive data from fixed sensors and transmit them to data centers. Zhao *et al*. [14] exploited human-carried or vehicle-mounted sensors to ubiquitously collect data and build various sensing maps. Packets are assumed to be spatial-temporal correlated in the forwarding process, and two cooperative forwarding schemes that use data fusion are proposed: Epidemic Routing with Fusion (ERF) and Binary Spray-and-Wait with Fusion (BSWF), where the number of samplings and transmission overhead were demonstrated to be greatly reduced.

The scheme proposed at this paper belongs to the type of *opportunistic* data gathering schemes, where both the ordinary nodes and data collector are mobile and the “Store-Carry-Forwarding” strategy is adopted for message transmissions when collecting the data. In our previous work [13], we have proposed a data gathering scheme called PDA in mobile wireless sensor networks where both the collector and the ordinary nodes move and contact opportunistically. PDA collects the network meta-data to generate a node contact graph, base on which it calculates a data gathering location and sojourn time. The data collector is then controlled to move to the location to collect the data, avoiding unnecessary message transmissions. The sojourn time allocation problem is also considered at [8], yet it assumes that ordinary nodes are stationary. Feng *et al*. [30] proposed the distance-aware replica adaptive data gathering protocol in delay tolerant mobile sensor networks, which cuts down the number of redundant replicas of messages and leverages the delivery probabilities of nodes as main routing metrics. Differing from the schemes mentioned at [8,13], we assume the data collector could move faster in ADG, yet with much smaller time length within a data gathering round. So the contact opportunities with other ordinary mobile nodes are fewer, shorter, and more opportunistic in nature, which makes the data gathering a challenging problem. ADG would select the proxy nodes, determine their visiting order and locations, and allocate time slots so that the collector could encounter the proxy mobile nodes and maximize the amount of gathered data from them.

## 3. Preliminaries

### 3.1. Network Model

**Figure 1:**
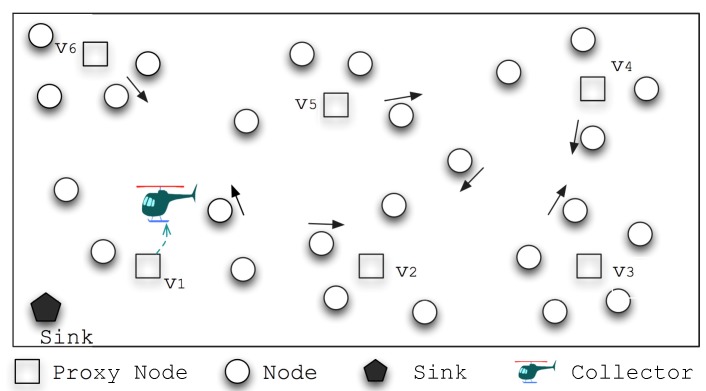
Illustration of data gathering using speedy mobile elements. Network meta-data are collected and extracted, based on which a set of proxy nodes are gathered. The data collector then follows the optimized schedule to pick up the sensed data from the proxy nodes through one hop transmissions, and then returns to the sink for data uploading.

There are *N* mobile nodes and one mobile data collector in the network. Every node has a unique id $s_{i}$ and senses data $d_{i}^{t}$ at time *t* from the environment. The data is stored at the node until collected by the mobile collector denoted as MC. Figure 1 is an example snapshot of the data gathering field. Ordinary nodes generate the sensed data, and some are selected as proxy nodes that act as intermedia storage that receive data from ordinary nodes. Data collector is feathered as relatively high moving speed, it collects data from the proxy nodes and then return back to the sink node for data uploading. We assume the network has the following characteristics:

1. Nodes adopt an opportunistic way for message transmission. Two nodes would establish a temporary communication link for message exchange when they are within each other’s communication range. The movement of nodes displays some kind of patterns in cycles during the epochs (e.g., one day/epoch), and there are some places of interest (POI) where nodes would get together and exchange messages with each other;
2. Mobile collector MC has no constraint on storage space. It is programmed and actuated by data gathering applications. MC starts its trip at the sink, moves within the network to gather the data; then the mobile collector has to return to the sink to upload the gathered data and replenish its energy. For a data gathering round, the time length of data gathering for a MC should be less than a threshold Υ;
3. Nodes could record their locations if needed, and they move much slower than the data collector. Data transfer from nodes to MC is only possible when they are both within each other’s communication range, and it reaches stable transmission when MC stays still and nodes move within the area of communication range.

The data gathering application is characterized by the time constraint for MC within a round of data gathering, which is denoted as Υ. As MC is controllable, ADG adopts optimization strategies to maximize the amount of gathered data. The proposed algorithm would calculate and set three factors for the optimization: (1) when should MC start its journey for the data gathering; (2) which nodes are going to be visited by MC; and (3) where and according to which order MC is going to visit the nodes and pick up the data.

### 3.2. Evaluation Metrics

Many factors have to be considered when designing a data gathering mechanism in the opportunistic sensor networks, yet in our model there are two basic evaluation metrics: *transmission overhead* and *data coverage*.

Transmission overhead indicates the efficiency and overall cost of the algorithm, it is represented by the total number of messages sent by the ordinary sensing nodes. Fewer transmissions mean less chance of congestion and conserve more energy, especially when considering most of mobile nodes are battery-powered. Data coverage indicates the effectiveness of the algorithm, which is defined by the amount of collected data divided by the total sensed data. Unlike other data gathering mechanisms in stationary wireless sensor network, the data gathering latency is not treated as a key metric for our algorithm. This is because we assume the network is delay-tolerant, and the maximal gathering latency is constrained by Υ, which is the duration of a data gathering round defined at the application.

## 4. Algorithm Description

### 4.1. Overview

ADG adopts an adaptive and schedule-based strategy for the data gathering at mobile wireless sensor networks. A round of data gathering task in ADG could be roughly divided into 4 phases:

1. **Meta-data Extraction**: nodes collect network meta-data to facilitate cooperative data collection. Each node extracts and calculates meta-data such as locations, number of contacts, contact duration, *etc*.
2. **Proxy Node Selection**: MC collects the meta-data, extracts parameters and calculates a set of proxy nodes. Proxy nodes act as intermediate storage, where other nodes would send their sensed data to them through opportunistic communications.
3. **Visiting Order Scheduling**: MC adopts a schedule-based strategy for the data gathering. The visiting schedule of proxy nodes includes three aspects: (a) when should MC start its journey for the data gathering; (b) which nodes are going to be visited by MC; and (c) where and according to which order MC is going to visit the nodes and pick up the data.
4. **Data Gathering from Proxy Nodes**: MC travels to each of the predicted data gathering points according to the schedule and collects the sensed data from the proxy nodes. When a round of data gathering ends, MC goes back to the sink area to upload the collected data and replenish its energy.

**Table 1:** Notation table.

Notation	Definition
$s_{i},v_{i}$	ordinary node, proxy node
$ar_{i,\Delta }$	activity range of $s_{i}$ within duration Δ
ss,sd	stationary stay, stationary duration; $ss=(sd,C_{sd})$
$C_{sd}$	the central location within sd for a node
kss,ksd	key stationary stay, key stationary duration; kss=(ksd,Ω)
Ω	possible locations and their weights during ksd for a node
*R*	communication range
*W*	observing window
*V*	set of proxy nodes
*E*	time line within an epoch
$e_{i}$	the *i*th epoch
$w_{i}$	weight of node for proxy selection
$\mu_{i}$	accumulated weight of stationary stay for $s_{i}$
$\eta_{i}$	number of distinct encounters for $s_{i}$
${ℵ}_{i}$	set of key stationary stays of node $s_{i}$
Φ	recorded location of node
Υ	data gathering period
Ψ	schedule of data gathering for MC
$\rho (v_{i})$	expected amount of data stored at $v_{i}$
$T_{slot}$	the minimal data gathering duration of a slot
th	predefined threshold for proxy node selection
$e(q_{k})$	index of epoch from which the central point $q_{k}$ is extracted
p1(x)	expected probability that x is within a stationary duration
$p2(s_{i},s_{j})$	encounter probability between $s_{i}$ and $s_{j}$
$p3(C_{Q})$	encounter probability of $C_{Q}$
$C_{Q},C_{Q^{*}}$	centroid of the set of points in *Q*, centroid point that has the largest encounter probability

**Figure 2:**
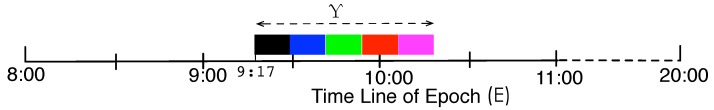
An example of data gathering schedule within an epoch.

Steps 2–4 are repeated as the data gathering task is epoch-based. The meta-data gathering and proxy node selection are preparation process for the coming of mobile data collector: data is routed and stored at the proxy nodes until MC comes and picks them up. Compared with other message forwarding protocols in MON, data messages in ADG have clearer targets—the proxy nodes, which have high probability to encounter MC and uploads their data when following the optimized data gathering schedule. Figure 2 illustrates an example of data gathering schedule within an epoch, where the length of a round of data gathering is 60 min (Υ) and the length of epoch is 12 h (8:00–20:00). According to the optimized schedule, MC would begin the data gathering trip at 9:17, and there are 5 nodes at the set of to-be-visited proxy nodes, where each time slot of visiting is denoted by the colored rectangle. The overall goal of ADG is to gather the maximal amount of data from nodes during the constrained time period at the right place, and to avoid redundant or long hops of message transmissions.

Table 1 lists the Notations and their definitions in the paper. In the following subsections, we present the detailed description of main phases of the algorithm.

### 4.2. Meta-Data Extraction

In ADG nodes record and extract the network meta-data to facilitate data gathering. Each node periodically records its location and encountered nodes, based on which the meta-data about the node activity and contacts could be derived.

#### 4.2.1. Activity Meta-Data

Suppose within a time interval $\Delta =[t_{1},t_{K}]$ the locations in the trajectory of node $s_{i}$ is $\Phi =\{l_{1},..,l_{K}\}$, where $l_{k}$ corresponds to the recorded location of node at time $t_{k}$. Then the *activity range* of node $s_{i}$ is defined as follows:

(1) $$ar_{i,\Delta }=max(||l_{k},C_{\Delta }||),\;k=1,..,K$$

where ||a,b|| denotes the Euclidean distance of point *a* and *b*, and $C_{\Delta }$ is the geometric center of the recorded points. The centroid $C_{\Delta }$ is defined as:

(2) $$C_{\Delta }=centroid\left(\Phi \right)=\left(\frac{1}{K}\sum_{k=1}^{K}l_{k}.x,\;\frac{1}{K}\sum_{k=1}^{K}l_{k}.y\right)$$

**Figure 3:**
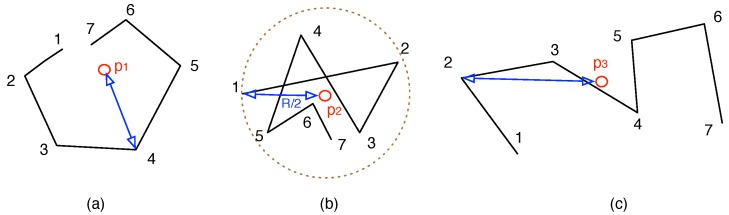
Examples of node trajectories and their activity ranges: (**a**) moving trajectories of node $s_{1}$; (**b**) moving trajectories of node $s_{2}$; (**c**) moving trajectories of node $s_{3}$.

Based on the activity range, ADG then calculates the time duration and the central point when a node stays stationary or only roams within a small area. We abstract these as the *stationary stay* (ss), which is defined as $\left(sd,C_{sd}\right)$. sd is the *stationary duration*, $C_{sd}$ is the central location within the duration. $sd=[t_{m},t_{n}]$ follows the following conditions:

(3) (1)tn-tm>τ,[tm,tn]⊆E;(2)ari,sd≤0.5R;(3)∃sd′⊃sd,s.t.ari,sd′≤0.5R

where *τ* is the minimal time length of stationary duration, *E* is the time range within an epoch, e.g., [8:00, 20:00], *R* is the communication range of the node. Here the activity range $ar_{i,sd}$ is less than half of the communication range *R*, so any two nodes, if they are both within the circular area centered at $C_{sd}$, could communicate with each other and makes the message exchange. Figure 3 illustrates examples of the trajectories and their average ranges. The lines are the moving trajectories of the nodes, the numbers denote the timestamps at that position, $p_{i}$ is the centroid and the line segments in blue color is the activity range ar. We could see that node $s_{2}$ has smallest ar, yet node $s_{3}$ has the largest ar. Also, if $ar_{2,[t_{1},t_{7}]}=0.5R$, then the stationary duration for node $s_{2}$ is $\left[t_{1},t_{7}\right]$, and any node within circle $p_{2}$ could communicate with $s_{2}$ during the time period.

**Figure 4:**
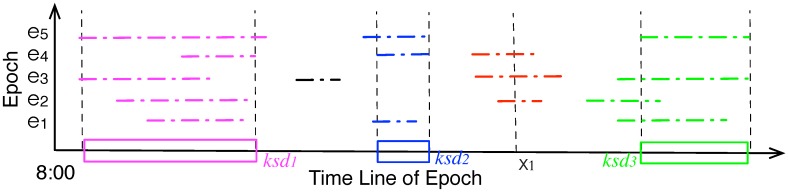
Mapping the maximal stationary durations (sd) into set of key stationary durations (ksd) with threshold θ=0.6.

A node usually has more than one stationary stays in an epoch, and it records multiple stationary stays after several epochs. Figure 4 illustrates an example of stationary durations during an observing window of 5 epochs. The x-axis is the line of epoch, e.g., 8:00 to 20:00, the y-axis denotes id of the epochs. The colored line segments are the static durations (sd) at different epochs, and sd’s from multiple epochs could be aggregated into the key static durations (ksd), which are denoted by the colored rectangles. Given an observing window *W*, the expected probability that a point, e.g., *x*, is within a stationary duration is estimated as:

(4) $$p1\left(x\right)=\sum_{e_{k}\in W}f\left(x,e_{k}\right)=\sum_{e_{k}\in W}\frac{sgn(x,e_{k})}{2^{|W|+1-k}}$$

where *f* is a weighting function, sgn is a function signifying 1 or 0. When time *x* is within a sd in epoch $e_{k}$, $sgn(x,e_{k})$=1, and the weight $f(x,e_{k})$ equals $\frac{1}{2^{|W|+1-k}}$; otherwise, $sgn(s_{i},s_{j},k,\Delta )$=0, and the weight $f(x,e_{k})$ is 0. We assume later sd has larger impact on the weight. Although various weight degrading functions could be used, here we use a simple one: the weight is divided by $2^{|W|+1-k}$, which means the weight of a sd is twice of that of the previous one. As illustrated at Figure 4, $x_{1}$ lies within a stationary duration at the 2nd, 3rd and 4th epochs, the estimated probability of $x_{1}$ is: $\sum_{i=1}^{5}f\left(x_{1},e_{i}\right)$=$f\left(x_{1},e_{2}\right)+f\left(x_{1},e_{3}\right)+f\left(x_{1},e_{4}\right)$=$\frac{1}{2^{5+1-2}}+\frac{1}{2^{5+1-3}}+\frac{1}{2^{5+1-4}}$ = 0.4375.

ADG then aggregates and maps the *stationary durations* (sd) into a set of *key stationary duration* (ksd). All points in the ksd has a estimated probability greater than a threshold parameter *θ*, and it means the node would stay stationary or only roams within a small area during the time interval ksd with probability higher than *θ*. A ksd should meet the following conditions:

(5) (1)∀x∈ksd,p(x)≥θ;(2)∃ksd′⊃ksd,s.t.∀y∈ksd′p(y)≥θ

Condition 2 means ksd is the maximal superset of the range, and it does not exist another key static duration that contains the ksd. Figure 4 also illustrates the set of $ksd^{\prime }s$ with θ=0.6 (is also the default value in simulation), and readers could refer to Appendix A for the detailed algorithm that extracts a nodes’s the $ksd^{\prime }s$.

A stationary stay is denoted as $ss=(sd,C_{sd})$, where sd is the stationary duration and $C_{sd}$ is the central point. Correspondingly, stationary stays are mapped into a *key stationary stay* (kss), and a *key stationary stay* is denoted as kss=(ksd,Ω). Here Ω denotes the possible locations and their weights the node might be in during the key stationary duration ksd.

A node might have multiple key stationary stays. The set of key stationary stays of node $s_{i}$ is denoted as ${ℵ}_{i}$, then the *accumulated weight of stationary stay* of node $s_{i}$ is denoted as $\mu_{i}$, which is a key factor for the proxy node selection:

(6) $$\mu_{i}=\sum_{kss\in {ℵ}_{i}}\left|kss.ksd\right|*{|{ℵ}_{i}|}^{\frac{1}{2}}$$

where |kss.ksd| denotes the length of key stationary duration in kss, $|{ℵ}_{i}|$ is the number of elements in the set. From the equation, we could see that $\mu_{i}$ goes up when the $ksd^{\prime }s$ have longer accumulated time length and are distributed to more separated segments on the time line of an epoch.

#### 4.2.2. Contact Meta-Data

Nodes also record their encountered nodes as *contact log* (CL). Tuple in CL is in the form of $\left(node\_id,t_{b},t_{e}\right)$, where node_id is the id of encountered node, $t_{b}$ and $t_{e}$ denote the beginning and the end of the contact respectively, and the contact duration $|t_{e}-t_{b}|$ should be greater than a predefined threshold. Based on the contact logs, the *number of distinct contacts* of $s_{i}$, denoted as $\eta_{i}$, could be calculated. *η* is a key parameter for proxy node selection. The larger *η* is, the more active the node would be, and the more suitable for the node to be a proxy node.

Also, ADG adopts an opportunistic strategy when forwarding messages in MON. A node, e.g., $s_{i}$, could calculate the encounter probability with any other node given an observing time window *W*:

(7) $$p2\left(s_{i},s_{j}\right)=\sum_{e_{k}\in W}g\left(s_{i},s_{j},e_{k}\right)=\sum_{e_{k}\in W}\frac{sgn(s_{i},s_{j},e_{k})}{2^{|W|+1-k}}$$

where *g* is a user-defined weighting function, sgn is a function signifying 1 or 0. When $s_{i}$ encounters $s_{j}$ at kth epoch, $sgn(s_{i},s_{j},e_{k})$=1, and the weight is $\frac{1}{2^{|W|+1-k}}$; otherwise, when they do not encounter each other, $sgn(s_{i},s_{j},e_{k})$=0, and the weight is 0. The calculation is similar with that in Equation (4), and we assume later contacts has greater impact on the weight. For example, when |W|=4, if node $s_{i}$ encounters $s_{j}$ at the 2nd and 4th epoch, the encounter probability $p(s_{i},s_{j},\Delta )$=$\frac{1}{2^{4+1-2}}+\frac{1}{2^{4+1-4}}$ = 0.625.

### 4.3. Proxy Node Selection

At initial epochs, nodes exchange meta-data with other nodes through opportunistic communications, e.g., epidemic routing [31]. When MC moves within the field, e.g., following the random waypoint mode, the neighboring nodes would send their sensed data and meta-data to the collector. After some time, MC would have accumulated enough meta-data for the selection of proxy nodes.

The proxy selection is based on the weight of a node, which is composed of two parts: the *accumulated weight of stationary stay*$\mu_{i}$ and the *number of distinct encounters* $\eta_{i}$:

(8) $$w\left(s_{i}\right)=\alpha *\frac{\mu_{i}}{\mu_{max}}+\left(1-\alpha \right)*\frac{\eta_{i}}{\eta_{max}}$$

where α∈[0,1] is the balance factor, $\mu_{i}$ is defined at Equation (6), $\eta_{i}$ is the distinct number of nodes that contact with node $s_{i}$. $\mu_{max}$ and $\eta_{max}$ are the maximal values of corresponding parameters MC has ever known. They could be extracted by MC through initial rounds of data gathering and meta-data exchanging. Parameter $\mu_{max}$ and $\eta_{max}$ are then broadcasted to the sensing field, and every node would use these parameters to calculate its weight. If $w(s_{i})$ is greater than a predefined threshold th, or a node is a proxy node in previous epoch yet does not upload its data to MC, then $s_{i}$ would promote itself as the proxy node at current epoch. Also, the number of proxy nodes could be controlled by the parameter settings. Proxy nodes act as the intermediate data storage as other nodes would forward their sensed data to them; then the mobile collector would come and pick up the data from the proxy nodes.

It is worth to be noted that given the $\mu_{max}$, $\eta_{max}$ and th, a node could determine whether it would act as a proxy node or not. If a node changes its state of role, e.g., to be newly promoted as a proxy node or turn back as a ordinary node, it would advertise itself by broadcasting a *state-change* message. In this way, other nodes and MC will be informed on that change. Also, although nodes are classified into *ordinary nodes* and *proxy nodes*, the network is not partitioned into clusters of subregions or subnetworks, e.g., a node is attached to some cluster and it only route its data to the cluster head. Instead, within the network all nodes are mobile, and they adopt an opportunistic way for message transmissions. When two nodes are in contact, they would establish a temporary communication link for message exchange. Ordinary nodes would forward their data to the proxy nodes whoever they encounter.

### 4.4. Visiting Order Scheduling

Once proxy nodes gather the sensed data from their neighboring nodes, these data should be collected by the mobile collector when they are in contact. Usually there are more than one proxy nodes, and MC has to arrange its visiting order and time slots so that it could gather the maximal amount of data within the limited period. The scheduling could also be viewed as the Proxy node Time Slot Allocation (PTSA) problem.

**PTSA Problem**: Given a set of proxy nodes V, find a set of proxy nodes $V^{*}=\left\{v_{1},..,v_{K}\right\}$ and their visiting schedule Ψ *during the data gathering period *Υ*, such that the expected amount of gathered data *χ* is maximized and the schedule *Ψ* is compatible.*

Firstly, the expected amount of gathered data by visiting the set of proxy nodes $V^{*}$ is estimated as:

(9) $$\chi =\sum_{v_{i}\in V^{*}}\rho \left(v_{i}\right)=\sum_{v_{i}\in V^{*}}\eta_{i}*A\left(r\right)$$

where $\rho (v_{i})$ is the expected amount of data stored at a proxy node, $\eta_{i}$ is the number of distinct contacts of $v_{i}$, A(r) denotes the amount of data a node might have in data gathering round *r*.

Secondly, when a scheduler is compatible, the collector would visit each of the nodes one by one within the data gathering period $\Upsilon =[b_{0},e_{K+1}]\subset E$, and would not conflict with each other. Formally, a schedule $\Psi =\{\left(v_{1},\left[b_{1},e_{1}\right]\right),..,\left(v_{i},\left[b_{i},e_{i}\right]\right),..\left(v_{K},\left[b_{K},e_{K}\right]\right)\}$ is said to be compatible if it follows the following condition:

(10) $$\forall \;\left(v_{i},\left[b_{i},e_{i}\right]\right)\in \Psi ,\;\;\;\left[b_{i},e_{i}\right]\subseteq SD\left(v_{i}\right),\;e_{i}-b_{i}\geq T_{slot},\;\;b_{0}\leq b_{i}<e_{i}<b_{i+1}\leq e_{K+1}$$

where $\left(v_{i},\left[b_{i},e_{i}\right]\right)$ denotes visiting $v_{i}$ during the time range $\left[b_{i},e_{i}\right]$, $SD(v_{i})$ denotes one of the key stationary duration of $v_{i}$, and $T_{slot}$ is minimal data gathering duration of a slot. Each slot is assigned to a proxy node for MC’s visiting, and they would not overlap with each other. Because the mobile collector could move fast, here we assume the time duration moving from one proxy node to the next is negligible compared with the period of data gathering round. So the collector MC is only constrained by the duration of data gathering, which is denoted by Υ.

**Figure 5:**
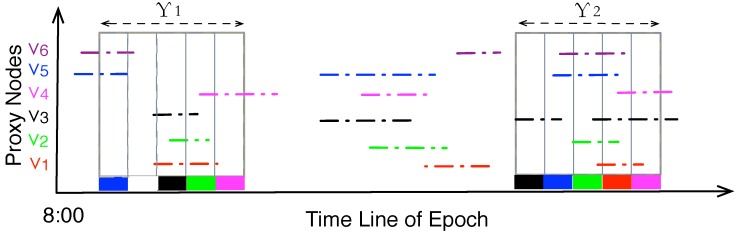
Mapping the Proxy node Time Slot Allocation (PTSA) problem into the problem of maximal coverage of line segment.

The PTSA problem is a variant of the famous “Knapsack problem” [20]: given a set of proxy nodes, each with an amount of data to be gathered, determine the set of proxy nodes to include in the data gathering period so that the total amount of data is as large as possible, under the constraints that the visiting of proxy nodes should be continuous and the *key stationary stays* should not overlap each other in the time dimension. This problem is also defined as the *maximal coverage of line segments*, where the covering line is the data gathering duration. In Figure 5, the x-axis is the line of epoch, e.g., 8:00 to 20:00, the y-axis denotes id of the proxy nodes. The colored line segments are the key static durations (ksd) of different proxy nodes. The key stationary durations ($ksd^{\prime }s$) are projected on the time line of epochs (x-axis ), and they overlap with each other. ADG then calculates a period of data gathering round with length Υ, and assigns each of its slots to a proxy node, so that the total weight of the slots is maximized. Here the weight of a time slot is the expected amount of data that could be gathered by the MC during that slot. When a proxy node *v* is assigned to a time slot *s*, MC is supposed to encounter *v* and gather its stored data, so the weight of *s* is the total amount of data stored at *v*. From the figure, we could see that $\Upsilon_{2}$ is preferred compared with $\Upsilon_{1}$ because it covers more slots, and hence might get more amount of data from the proxy nodes. Note that the duration Υ could only cover a slot exactly once because within an exact time slot MC could only visit one proxy node, and hence could prune large searching space. Readers could refer to Appendix B for the detailed algorithms that solves the PTSA problem.

It is possible that some proxy nodes, e.g., $v_{6}$ in Figure 5, might not be visited by MC within a gathering round. In ADG, the unvisited proxy node would double its weight A(r) at the next round of data gathering, meaning more data is stored at the node. So at the next epoch the unvisited proxy node would be added to the *to-be-visited* set of nodes according to the algorithm, and the data could be picked up by the data collector.

### 4.5. Data Gathering from Proxy Nodes

When a new data gathering round begins, MC would visit the proxy nodes one by one according to schedule Ψ. When they are in contact, the sensing data that have been routed and stored at the proxy nodes are gathered by MC. Proxy nodes has high probability to be stationary and hence be able to make a stable connection for data transfer. Yet MC still has to figure out the location of the proxy node in order to visit a proxy node during its key stationary stay (kss) according to the schedule.

For a proxy node *v*, suppose the key stationary stay kss is mapped from *m* stationary stays from *m* epochs, where each stationary stay is composed of a stationary duration sd and the central point $C_{sd}$; then the set of *m* central points are aggregated according to their locations. A set of central points, e.g., $Q=\{q_{1},..,q_{k}\}$, could be aggregated into a new point if they satisfy the following conditions:

(11) $$k=1,\;\;\;or\;\;distance(q_{i},q_{j})\leq 2R,\;\;\;i,j=1,..,k$$

where *R* is the radius of communication range. Points in *Q* could then represented by a new point: $C_{Q}=centroid\left(Q\right)$, which is defined at Equation (3). Similar with the expected probability of a point defined at Equation (4), the *encounter probability* of $C_{Q}$ is then defined as:

(12) $$p3\left(C_{Q}\right)=\sum_{q_{k}\in Q}h\left(q_{k}\right)=\sum_{q_{k}\in Q}\frac{1}{2^{\left|W|+1-e(q_{k}\right)}}$$

where $e(q_{k})$ is the index of epoch from which the central point $q_{k}$ is extracted. The data gathering point for proxy node *v* is then set to be the point who has the largest encounter probability, denote as $C_{Q^{*}}$. MC then just moves to point $C_{Q^{*}}$ and prepares for encountering the proxy node and gathers the data.

Although the node movement is assumed to roughly follow some kind of pattern within the network, and nodes would stay at some area during the key stationary duration with high probability, it is also possible that a proxy node deviates from the expected location and moves to other area. At this case the proxy node would periodically broadcast its locations to its neighboring nodes so that MC is able to get the proxy node’s exact locations from neighboring nodes, and move to the new location to pick up the data during the data gathering time slot. MC also collects data from ordinary nodes when they are in the communication range, and gathers the network meta-data for proxy nodes selection and time slot allocation in the next epoch.

## 5. Experimental Section

### 5.1. Environment Setup

We implement ADG in C# and compare it with other data gathering schemes. As nodes are mobile, we assume relatively sparse deployment of nodes for the wireless sensor network: there are 36 nodes within a rectanglar area, and the network is divided into 8*8 grids. The community model described in [32] is adapted to simulate the movement of nodes, where each grid is a community and each community has an interest index $c_{i}\in \left(0,1\right)$. If the index is greater than a threshold, the community is called a *Hot Community*, and the set of hot communities in the network is denoted as *C*. At the beginning of the simulation, each node is tagged as *ordinary node*, and each ordinary node is assigned an id, e.g., $s_{i}$, and an grid as its *home community*. Each node starts its movement from its home community, moves along its path, and then goes back to the home community. Each node moves according to a set of paths, and each path is composed of several communities, including the hot communities. A node stops and moves within community $c_{i}\in C$ for a period with probability of $P_{stop}=ps+\left(1-ps\right)*a_{i}$; and chooses to move to the next community along the path with probability $1-P_{stop}$, where ps is the predefined value, $a_{i}$ is the interest index of the target community. $\left(1-ps\right)*a_{i}$ represents the probability that a node would stop because of the distraction within the community that it currently visits. A node has several predefined paths, and the path to move along is randomly chosen from them. An ordinary node may promote itself as a proxy node and change its tag to *proxy node* according to Section 4.3, yet this has no impact the node movements. The MC is not active until a data gathering round is fired. It moves to the predicted location of proxy nodes one by one according to the optimized schedule, stop at each location for a period of time expecting to receive data from the proxy nodes. Table 2 summarizes the default values of network model and parameters in the simulation.

The simulation runs periodically. It consists of 30 epochs, where each epoch has a period of 14,400 simulation seconds (s), and the length of data a gathering round (Υ) is 1800 s. For the sensed data, every node adopts a Poisson process to trigger an event for data generation, where the interval between two sequential events follows the Poisson distribution with parameter *λ* = 600 s. The size of date generated by an event is 64 K, so the expected amount of total sensing data generated by a network of 36 nodes is 54 M (36*64 K*14400/600) per epoch. The bandwidth of link that uploads data from ordinary nodes to MC is 64 KBps. We assume ideal links when two nodes meet and establish a connection.

ADG is a data gathering scheme that adopts speedy mobile elements at opportunistic mobile sensor networks under limited data gathering period. It is worth noted that “opportunistic forwarding” and “limited data gathering period” are the two main characteristics of the proposed ADG scheme compared with other data gathering schemes. Ordinary nodes would move to other places, and tracks could not be predefined or calculated for the data collector. So the track-base or roaming-based data gathering schemes [3,4,6] could not be directly adopted for the data gathering scenarios of opportunistic mobile sensor networks. For fair and extensive comparison of the proposed algorithm, we implemented other four data gathering schemes that adopt opportunistic forwarding or proxy based strategies:

- (1) Epidemic [31]: MC moves according to the way-point mobility model, yet nodes take advantage of all chances of communications, data are exchanged among any nodes if possible and finally gathered by the MC;
- (2) PROPHET [32]: MC moves as the same pattern in Epidemic, yet data are exchanged according to the data forwarding probability based on the movement history;
- (3) PDA [13]: a node contact graph is created to compute the data gathering location where MC could contact with more nodes during some time period; MC is then programmed to move to the gathering point to gather the data;
- (4) PROXY: nodes with more contacts are promoted as proxy nodes. Proxy nodes broadcast their location information to notify neighboring nodes for opportunistic data collection, and MC would visit the proxy nodes according to a scheduled trajectory. The trajectory planning is similar to reference [25] based on the solution of the Traveling Salesman Problem.

**Table 2:** Default parameters of the simulations.

Parameter	Value	Description
*N*	36	number of nodes
field	800*800 m${}^{2}$	area of the sensing field
grid	10*10	grid partition of the field
i_n,t_n	5,30	initial and total number of epochs
|epoch|	$1.44*10^{4}$ s	length of an epoch
|W|	8	number of epochs in observing window
Υ	1800 s	data gathering round for MC
slot_n	9	number of slots (200 s/slot)
sp1,sp2	[2, 4], [10, 20] m/s	node and MC’ range of speed
ps	∼N(0.2,0.1)	nodes’ basic probability that stops at a grid
sd	∼N(360,60)	nodes’ stop duration at a grid
*C*	24,31,36,45, 50,54,67,76,83	set of hot communities
hot_r	[1,3]	rang of number of hot communities for a path
path_m	2	maximal number of paths a node has
gird_r	[4,10]	range of number of grids in a path
*α*	0.5	balance factor at Equation (8)
th	0.4	threshold for proxy node selection
cache_s	128 M	size of cache for a node
packet_s	1 K	size of a packet
*R*	30 m	nodes’ communication range
*B*	64 KBps	bandwidth for communication
event_s	64 K	size of date generated by an event
*λ*	600 s	Poisson parameter for events

Epidemic and PROPHET are two classic routing protocols for opportunistic data forwarding; PDA is based on the opportunistic forwarding and contact graph; PROXY is close to ADG in that ordinary nodes route their data to the proxy nodes using opportunistic forwarding schemes, e.g., the Spray and Wait scheme [33]. The differences lies in that in PROXY the selection of proxy nodes does not consider the mode of node movements (e.g., stationary stay, contact duration), and proxy nodes have to proactively broadcast their locations to notify MC when MC schedules its path to gather data from the proxy nodes.

### 5.2. Overall Performance

In Table 3, row 1 lists the average number of messages of meta-data exchange, row 2 lists the number of messages that upload data to MC, row 3 lists the number of total messages per epoch, row 4 lists the *data coverage*, and row 5 lists the *efficiency* of the algorithms. The results presented at each cell is the average of 6 rounds of simulations, given the default parameters such as number of nodes, the data gathering period, and etc. At the simulation all the nodes are mobile, and they take advantage of the opportunistic communication chances for the data gathering. So each node is randomly assigned to a home community at each simulation and has its own paths, where the paths are different from one simulation to another.

For the overhead of message transmissions, all schemes except Epidemic incurs the cost of meta-data exchange and gathering. The number of messages for meta-data exchange is low, and it accounts for less than 15% of the overall message transmissions. Epidemic has no overhead of meta data exchange, yet it has the largest message transmission, as high as about 4.52E+5. This is because messages are exchanged among all the ordinary nodes; while at other schemes messages are selectively forwarded. At PROPHET, messages are forwarded to nodes who have larger probability to meet the MC, and at other schemes messages are only forwarded to the proxy nodes, saving lots of in-network message transmissions. So the overall data transmissions of PDA, PROXY and ADG are relatively small, which is more than 40% less than that of Epidemic. The amount of data upload messages is directly related to the data coverage. ADG has the largest data coverage, so the number of messages that upload the data to MC is the largest. MC receives about 3.99E+4 messages of sensed data from other nodes.

**Table 3:** Comparison of the overall performances.

Id	Metric	Epidemic	PROPHET	PDA	PROXY	ADG
1	*Meta Exchange*	0	1.83E+4	2.89E+4	2.94E+4	2.62E+4
2	*Data Upload*	5.77E+3	4.80E+3	1.97E+4	2.85E+4	3.99E+4
3	*Total Mssage*	4.52E+5	3.39E+5	1.85E+5	2.16E+5	1.78E+5
4	*Data Coverage*	10.43%	8.45%	35.64%	51.50%	72.12%
5	*Efficiency*	4.88%	5.02%	28.36%	32.62%	43.21%

*Data coverage* is defined as the amount of collected data divided by that of the total sensed data. From the table we could see that ADG has the largest data coverage, it gathers about 72.12% of the sensed data; while PROXY and PDA gathers 51.5% and 35.64% on average. The data coverage at ADG is about 6–8 times of those in Epidemic and PROPHET. MC at Epidemic and PROPHET could only collect data from whoever it encounters. Most of the data are not gathered because the data gathering period is relatively short, e.g., 1800 s. Instead, PDA, PROXY and ADG selectively compute the data gathering points; they guarantee a possible sojourn location that more data could be picked up by the MC. For PDA, the data gathering location is a place where more nodes get together; while in PROXY and ADG the data gathering locations are a set of proxy nodes, which are visited by MC for data gathering. However, due to the movement of proxy nodes, at the PROXY scheme MC might lose the track of the proxy nodes. At this case, gathering data from proxy nodes might not be feasible. At the ADG scheme, the sojourn location and time for the proxy nodes are carefully calculated, so MC would encounter the proxy nodes and picks up the data with high probability, leading to higher data coverage.

Metric *efficiency* is defined as the number of valued messages divided by the number of total messages. Messages are defined as valued if they are related with successfully uploaded data. They are the messages that are sent from the source node, forwarded among intermediate nodes, and successfully uploaded to MC. *Efficiency* is as high as 43.21% in ADG, while they are 4.88%, 5.02%, 28.36%, and 32.26% for Epidemic, PROPHET, PDA and PROXY respectively. This means more than 90 percent of the message transmissions at Epidemic and PROPHET do not lead to final successful data upload to MC; at other schemes the data gathering locations are carefully calculated, and there are proxy nodes for intermediate data storage, so more sensed data could be uploaded to MC and hence have higher efficiency.

### 5.3. Impact Factors Analysis

From the overall performance analysis we could see that ADG has great improvement compared with other schemes on the data coverage and overhead of message transmissions. ADG is specially suitable for data gathering at mobile sensor networks under limited data gathering period. The advantage lies in the careful selection of proxy nodes and their best stationary period so that there exists stable communication link for MC to pick up data from these nodes. In this subsection, we vary the basic network parameters to study their impacts on the performances of data gathering schemes.

#### 5.3.1. Network Density

**Figure 6:**
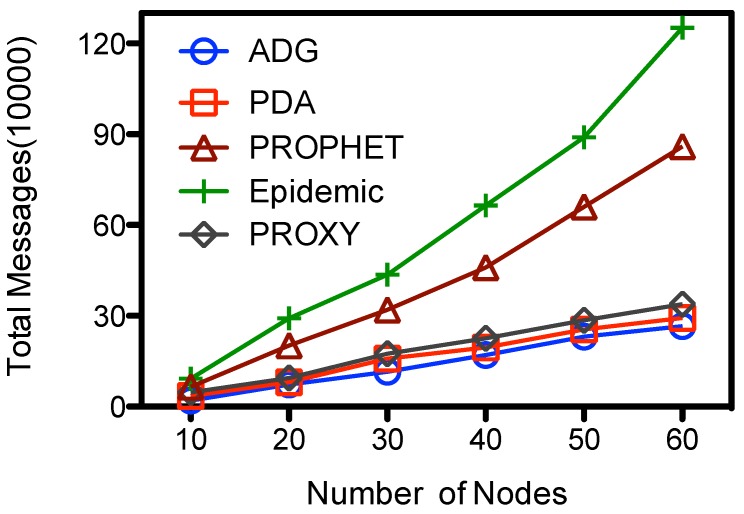
Number of nodes *vs.* total message transmissions.

From Figure 6 we could see that the number of message transmissions of all schemes goes up with the number of nodes. The Epidemic has the largest message transmissions, the number increases from 6.54E+4 to about 1.25E+6. Messages are exchanged among any two encountered nodes at Epidemic, while in other schemes the messages are selectively forwarded among the nodes. At the PDA, PROXY and ADG schemes messages are first selectively forwarded towards the proxy nodes, and then picked up by the MC. The former dominates the total cost of message transmissions, so the number of total message transmissions are very close. They have the lowest total message transmissions of the compared schemes, and the number is less than 25% of that in Epidemic when there are 60 nodes at the network. The performance of PROPHET lies in the middle, the number of transmissions is about 64%–70% of that at Epidemic at our simulation setting. For the proposed ADG scheme, ordinary nodes would sent their sensed data to the proxy nodes before the data are picked up by MC. There would be fewer messages exchanges among ordinary nodes in a sparse network because nodes would have fewer encounters. When there are more nodes, e.g., 60, the message transmission would goes up to about 2.65*E+5 at ADG, which is about 13 times of that when there are 10 node.

**Figure 7:**
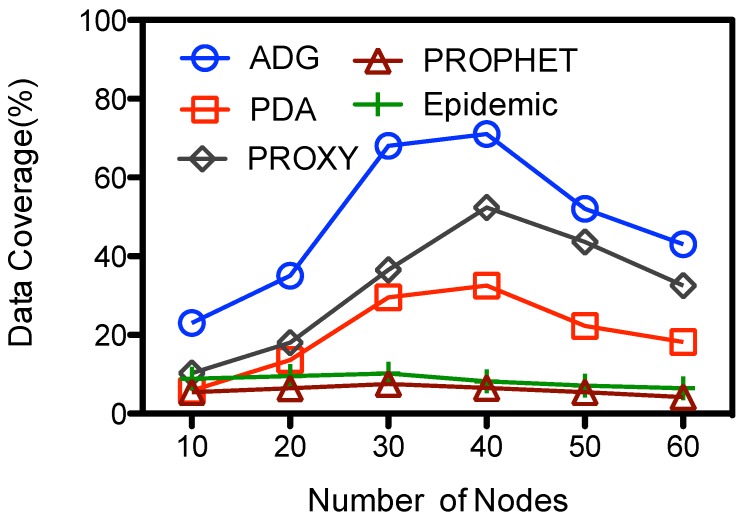
Number of nodes *vs.* data coverage.

Figure 7 shows the impact of network density to the data coverage. Epidemic and PROPHET has the smallest data gathering rate. Their data coverage decreases a little bit with the number of nodes, and the overall data coverage is less than 10.2%. This is mainly because of the limited data gathering period, which is about 12.5% of the total epoch. For other schemes their data coverage firstly goes up with the number of nodes, and reaches as high as about 72%, 52% and 32.5% for ADG, PDA, and PROXY respectively when 40 nodes are deployed. Proxy nodes would have more contact opportunities and receive data from ordinary nodes when there are more nodes within the network at these schemes. Yet ADG has higher data coverage because MC could collect the data from a set of proxy nodes while MC has only one data gathering location at PDA. Although there are multiple proxy nodes at the PROXY scheme, MC might lose the track of the proxy nodes as they keeps moving within the data gathering period. So the data at some proxy nodes could not be upload to the MC, which harms the data coverage. For the ADG scheme, when there are more nodes in the network, e.g., more than 40 nodes, there would be too many proxy nodes that act as intermediate storage to be visited by the MC. However, because the data gathering period is limited due to energy concern, MC is not able to collect all the data from the proxy nodes at one epoch, which decreases the data coverage. The data coverage is about 43% when there are 60 nodes and with one data collector.

**Figure 8:**
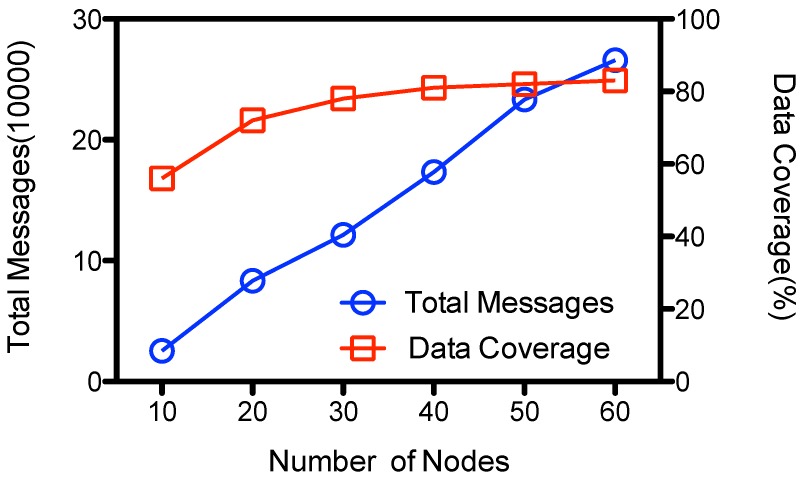
Performance of ADG with 2 MC.

However, it is easy to extend ADG using multiple collectors, or having multiple data gathering rounds at each epoch to handle the scalability problem of the data gathering. Figure 8 shows the performance results that use two data collectors. The amount of total transmissions is roughly the same with that at Figure 6, but the data coverage increases with the number of nodes due to the increased chance of contacts and meta-data exchanges within the network. It gathers as high as 82 percent of the sensed data when there are 60 nodes in the network. Generally, the number of collectors and the number of data gathering rounds could be tuned according to the network density and the data generating rate of the network.

#### 5.3.2. Data Gathering Period

**Figure 9:**
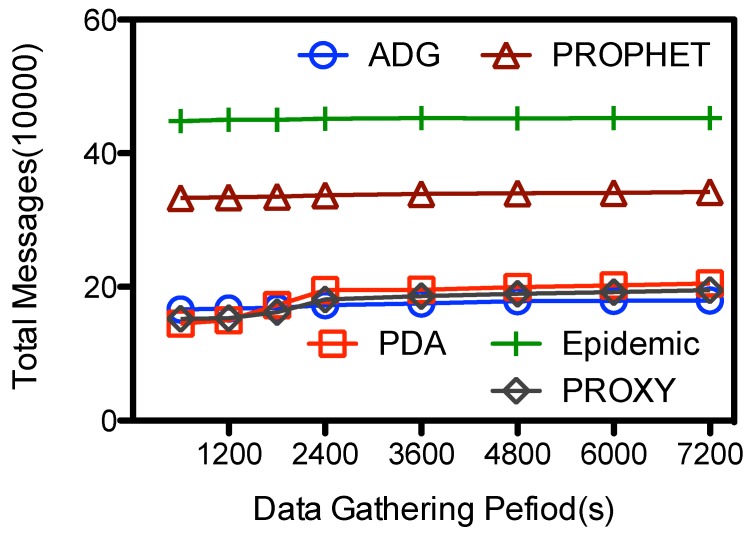
Data gathering duration (Υ) *vs.* total message transmissions.

Another impact factor is the length of data gathering period Υ, which is the period when MC are within the network. From Figure 9 we could see that the lines are relatively flat, showing that Υ has small impact on the number of total message transmissions. Larger Υ denotes larger contact chances between the mobile collector and in-network nodes and hence more message transmissions between them. The number of message transmissions are expected to goes up with Υ. When Υ increase from 600 to 7200 s, the number of total transmissions goes up from 1.66E+5 to 1.79E+5 for ADG, and from 1.45E+5 to 20.5E+5 for PDA. As the number of messages are mainly composed of in-network node-to-node transmissions, the impact of the increased message transmissions between MC and in-network nodes is relatively small on the overall messages transmissions.

**Figure 10:**
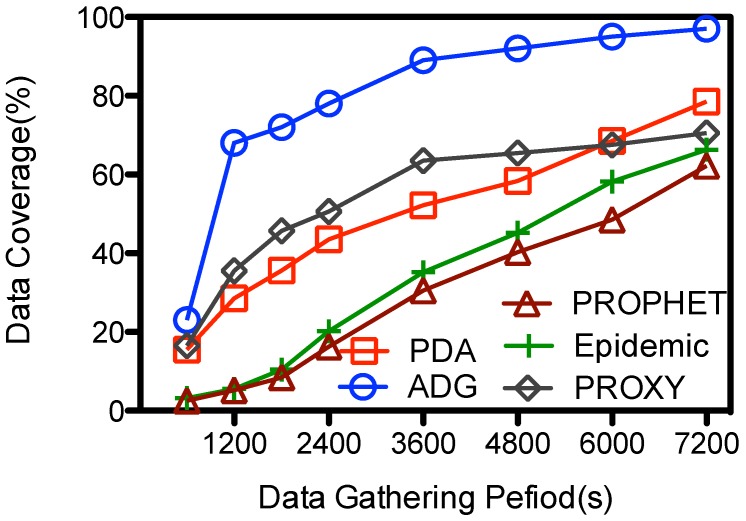
Data gathering duration (Υ) *vs.* data coverage.

The data coverage in all the schemes goes up with Υ, as illustrated in Figure 10. The PDA, PROPHET, Epidemic achieve their best performance at 78.5%, 66.3%, and 62.2% when the period is 7200 s; and their data coverage is less than 15% when the data gathering is limited and short, e.g., 600 s. These schemes are designed for general purpose routing and data gathering when the data gathering period is unlimited. So MC might not have enough time to contact with the in-network nodes and gathers the data from them when the data gathering period is small. However, the data coverage goes up relatively sharp from16% to 40% and from 20% to 62% for the PROXY and ADG schemes respectively when Υ increase from 600 to 1200 s, and then increases to near 71% and 93% respectively when the data gathering period is about 7200 s. Both schemes use proxy nodes as the intermediate storage. However, the proxy nodes at PROXY might still be moving when the MC is scheduled to collect the data from them, which makes the data transmission from some proxy nodes and MC unfeasible. Instead, at the ADG scheme the proxy node is scheduled to be visited during its *key stationary duration*, so message transmissions from the proxy node to the MC is stable and efficient. Given a fixed time slot for each proxy node, longer data gathering period means MC could visit more proxy nodes and hence gather more data from them. So when Υ is large, e.g., more than 6000 s, MC would have enough time to visit each proxy node several times and pick the data up, so the data coverage goes higher. Yet in real network deployment, the data gathering period Υ is limited because the speedy MC has a large energy consumption rate, and it should replenish its energy after a trip.

### 5.4. Impact Analysis for the ADG Scheme

Beside the basic network parameters, there are other designing factors in the proposed ADG scheme. At this subsection we study the impact of these factors, including the number of time slots, the threshold for proxy node selection, the change of node movement, and the imbalance of message transmissions.

#### 5.4.1. Threshold for Proxy Node Selection

**Figure 11:**
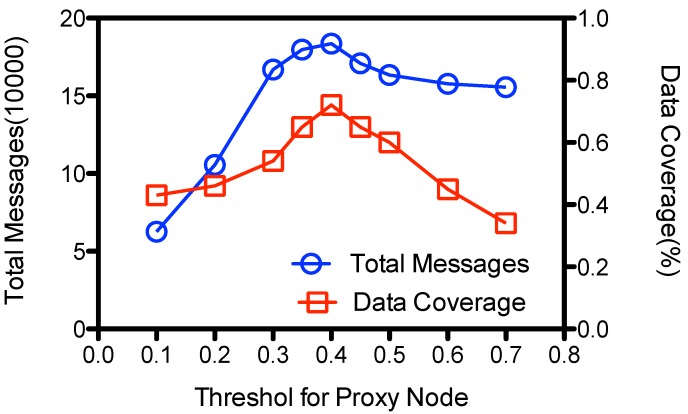
Impact of threshold for proxy node selection(th).

Proxy nodes are selected according to nodes’ weight. According to Equation (8), a node is promoted to be a proxy node if the weight is larger than a threshold th. As showed at Figure 11, both the number of total messages transmissions and data coverage goes up to the maximal when th is around 0.4, and then goes down as the threshold increases. When th is small, e.g., 0.1, most the nodes are promoted as proxy nodes, so they just keep the data and avoid lots of message forwarding; yet MC could not visit all the proxy nodes at the epochs, leaving some proxy nodes unvisited and some data un-gathered by MC, so the data coverage is low. When th is large, e.g., 0.6, fewer proxy nodes are selected, so there are fewer contact chances to upload its data to proxy nodes. This also leads to some un-gathered data at the ordinary nodes and makes the data coverage low. However, at this case the ordinary nodes would still try to forward their messages to the encountered nodes in order to be stored at the proxy nodes. So the number of total message transmissions is also high; more than 1.55E+5 messages are transmitted when th is more than 0.6. ADG achieves its best performance on data coverage at about 72% when th is within the range of [0.35, 0.5] and the number of message transmissions is about1.83E+5 at our simulation setting.

#### 5.4.2. Change of Node Movement

**Figure 12:**
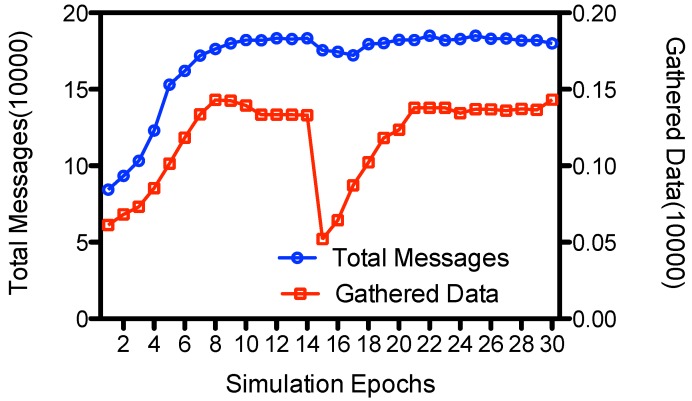
Message transmissions at each epoch.

ADG gathers the meta-data and adaptively selects the proxy nodes for the data gathering. So we changed the paths of network nodes at the middle of the simulation (at the15th epoch), and studied its impact on the amount of gathered data. Figure 12 depicts the average number of total messages and amount of gathered data at each epoch in 10 rounds of simulation. We could see that the number of gathered messages is small (less than 700 messages) at initial epochs, *i.e*., from 1 to 5 epochs. This is because at initial epochs nodes exchange meta-data with other nodes through opportunistic communications. The proxy nodes has not been selected, and the only way MC gathers data is that it would pick up data from its neighboring nodes when it moves within the field following a random waypoint mode. Yet after some epochs it gathers enough metadata and the proxy nodes are selected. During these epochs, nodes send their messages to proxy nodes, and MC visit the proxy nodes to gather the data. So both the total messages and the gathered data would increase. MC would gather about 1306 messages of sensed data at each round on average. When the node change their paths at epoch 15, we could see a sharp decline to about 521 messages on the number of gathered data. This is because MC would not encounter the proxy nodes when it visits the predicted location, since the proxy nodes have changed their paths and would not appear there. However, the proxy nodes would broadcast its new locations to neighbors; and after some epochs of meta-data gathering, new proxy nodes would be selected, and MC would acquire the new data gathering points of the proxy nodes. In this way, ADG adaptively updates the data gathering points and MC would again gather normal amount of sensed data from the proxy nodes. The data coverage returns to about 70.8%, which is almost the same as the case when the movement of nodes do not change.

#### 5.4.3. Imbalance of Message Transmissions

**Figure 13:**
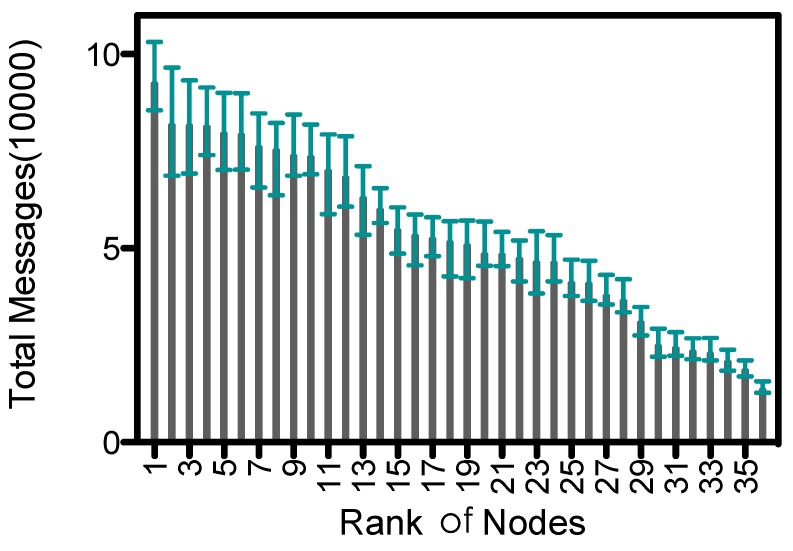
Message transmissions of nodes in descending order.

Proxy nodes receive data from ordinary nodes and upload them to the collector. So they have more message transmissions than ordinary nodes, which leads to an imbalance of energy consumption within the network. Figure 13 shows the descending order of average number of message transmissions for nodes in 6 rounds of simulation; the upper and lower bounds are also shown. The average number of messages is about 5.26E+4 , and standard variance is 2.18E+4. In ADG proxy nodes could be recalculated and updated among epochs, which helps balance the energy consumption. However, the maximal number of message transmissions is about 6.9 times of the minimal, which would lead to early exhaustion of energy at some proxy nodes. Yet recently, the breakthrough at wireless power transfer [34] has brought up a new possibility that MC would recharge the proxy nodes at the same time when proxy nodes are uploading the data. We leave the discussion of gathering data from rechargeable nodes as our future work.

## 6. Conclusions

In this paper, we have proposed a new scheme called ADG in mobile wireless sensor networks that adopts speedy mobile elements for data gathering. It collects the network meta-data for the selection of proxy nodes, and then solves a Proxy node Time Slot Allocation problem to calculate the orders and time slots according to which the collector visits the proxy nodes for data gathering. ADG fully takes advantage of the node movement patterns for the selection of proxy nodes and data gathering points, under the constraint of limited data gathering period. Also, the high speed of mobile element is exploited, and predefined paths or tracks are not needed. ADG is the first step on the search of a data gathering scheme using speedy mobile elements under the constraint of limited data gathering period, which brings about the potential of unmanned remote sensing in dangerous areas. Experimental results demonstrate that the proposed algorithm can greatly improve the data coverage in mobile sensing networks under the constraint of limited data gathering period.

In this work we assume loose time delay for the data gathering in mobile sensor networks, yet in some data gathering applications there might be a deadline for the data to be gathered. So in the future work we are going to find solutions of gathering data with deadlines in mobile sensor networks. Also, we are considering the possibility of recharging the proxy nodes through wireless power transfer by the mobile collector while they are in contact and uploading the sensed data.

## Appendix

### A. Algorithm of ksd Extraction

Here we maps the stationary durations (sd) into a set of key stationary durations (ksd). All points in the ksd should have a weight greater than a threshold parameter *θ*. Algorithm A1 presents the pseudocode of the key stationary duration extraction. Suppose there are *n* stationary durations, there are at most 2n points on the set ps, hence the complexity of the algorithm is O(2n+nlog(n)), where nlog(n) is the cost of sorting.

**Algorithm A1**: Key stationary duration extraction

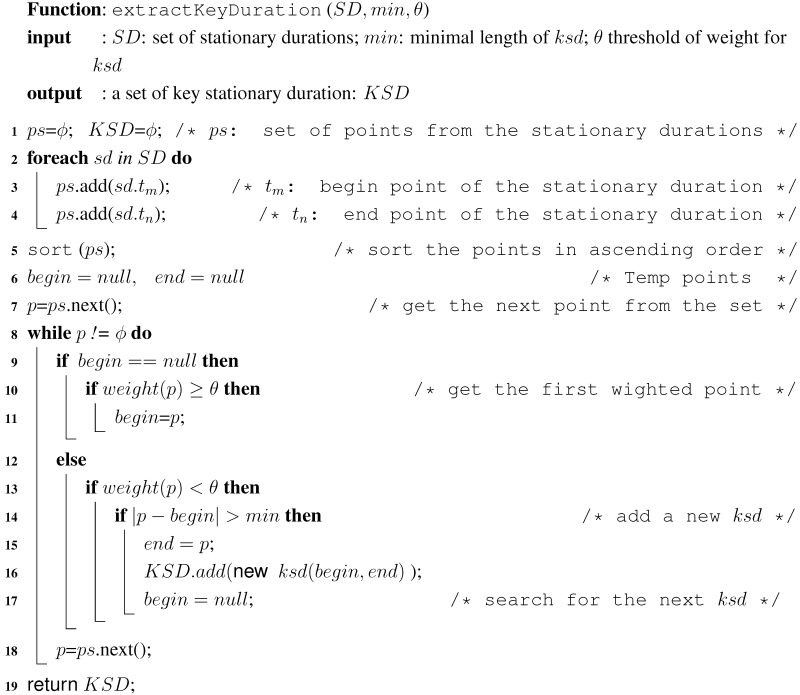

### B. Algorithm of Time Allocation for the Proxy Nodes

Algorithms B1 and B2 present the pseudocode of the proxy nodes time allocation. As described previously in Section 4.4, the allocation is transformed into the problem of *maximal coverage of line segment*, a variant of the famous “Knapsack problem” [20]. Suppose there are *n* key stationary durations ($ksd^{\prime }s$), and they are projected on the time line of epochs (x-axis ), so there are 2n points at ps. For each point, the algorithm calculates its maximal expected amount of data and node assignment through function getAmount. We denote *K* as the number of slots within a data gathering round, and each slot could be assigned to *z* proxy nodes at most, then the calculation space is ${\left(1+z\right)}^{k}$. So the overall time complexity is $O(2n*z^{K}$). Yet some conditions cuts down the solution space: (1) *K* is usually small, e.g., smaller than 10; (2) a proxy node could only be assigned to exactly one time slot; and (3) z<<n, and most slots are covered only by a few key durations.

**Algorithm B1**: Proxy node time allocation

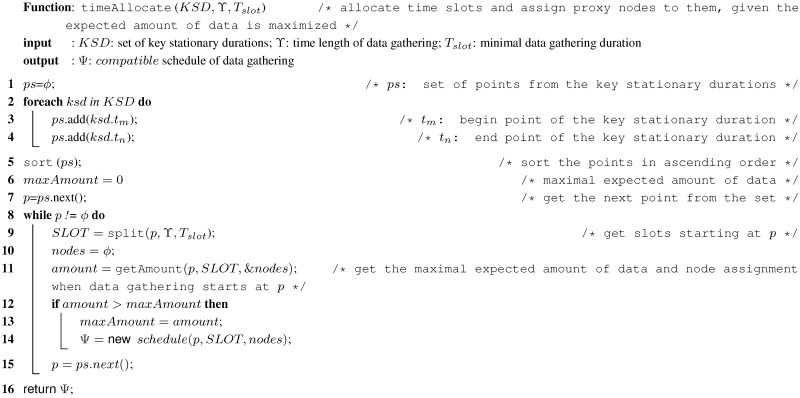

**Algorithm B2**: Get expected amount of data
